# Beast3D: Animal behavioral analysis and neural encoding from multi-view video via Gaussian splatting

**Published:** 2026-06-01

**Authors:** Yanchen Wang, Lenny Aharon, Wangshu Zhu, Kyle Daruwalla, Linghua Zhang, Jiaru Zou, Selmaan Chettih, Helen Hou, Liam Paninski, Matthew R Whiteway

**Affiliations:** Columbia University; Columbia University; Columbia University; Cold Spring Harbor; Cold Spring Harbor; Stanford University; Columbia University; Cold Spring Harbor; Columbia University; Columbia University

## Abstract

Multi-view video recordings are increasingly used to capture the 3D movements of animals in experimental settings, yet extracting rich 3D representations from these recordings remains challenging. Supervised pose estimation requires extensive manual annotation, while general-purpose 3D reconstruction models trained on generic scene datasets fail on the specialized imagery and sparse-view setting of laboratory experiments. We address these limitations with Beast3D, a self-supervised pretraining framework that learns 3D visual representations from unlabeled, calibrated multi-view video. Beast3D uses a vision transformer to predict 3D Gaussian splats that reconstruct held-out views through differentiable rendering, while simultaneously segmenting the animal from the background. Beast3D reconstructs 3D structure with as few as four views by conditioning directly on known camera parameters—unlike general-purpose models, which must estimate camera geometry from dense overlapping viewpoints that are seldom available in lab settings. Through comprehensive evaluation across four species, we demonstrate that Beast3D produces rich, viewpoint-invariant features that transfer effectively to three downstream tasks: *novel view synthesis*, which validates the quality of the learned 3D representations; *multi-view pose estimation*, which provides the sparse keypoint trajectories widely used in behavioral analysis; and *neural encoding*, which relates 3D behavioral features to simultaneously recorded neural activity. Beast3D thus establishes a versatile framework for behavioral analysis that leverages 3D structure in modern multi-view laboratory recordings. Our project page and code: https://ppwangyc.github.io/projects/beast3d.

## 1 Introduction

Advances in neuroscience increasingly depend on precise quantification of animal movement, yet the single-view video recordings that dominate current practice are fundamentally limited: self-occlusions obscure body parts, and 2D observations cannot recover the full 3D kinematics of movement [1]. A growing number of laboratories address these limitations by recording with multiple synchronized and calibrated cameras, enabling accurate 3D measurements of constrained and freely moving animals [2, 3, 4, 5, 6]. The resulting multi-view data support a range of 3D analysis tasks, from sparse keypoint estimation that describes posture and joint kinematics [7, 8, 9, 10, 11, 12, 13, 14] to dense 3D meshes that capture the full surface geometry and articulation of the subject [15, 16, 17]. These explicit 3D representations offer a natural basis for relating behavior to neural activity, providing an interpretable and low-dimensional description of animal movement independent of camera viewpoint.

**Figure 1:**
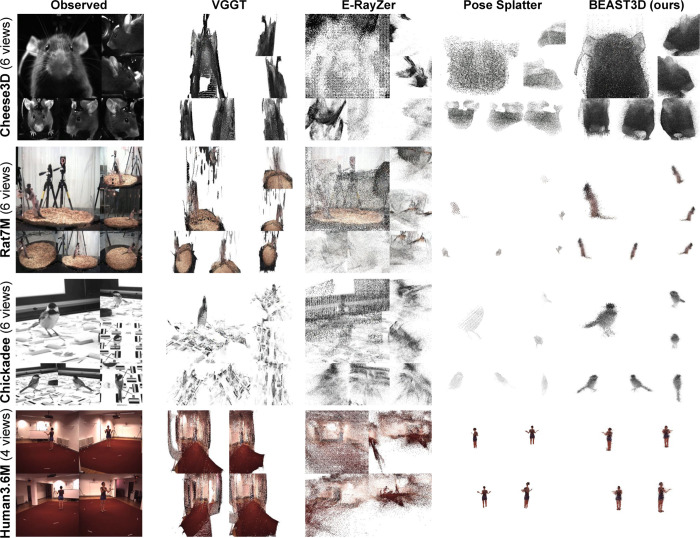
3D point clouds from Beast3D and leading baselines. An example scene from diverse datasets (*left* column; Cheese3D [18], Rat7M [2], Chickadee [3], Human3.6M [19]) is encoded into a 3D point cloud by general-purpose models (VGGT [20], E-RayZer [21]) and tailored per-dataset models (Pose Splatter [22], Beast3D). Beast3D achieves strong performance while simultaneously providing foreground segmentation of the subject. The point cloud positions are predicted by each model, and each point is colored by the corresponding color on each pixel.

Despite the richness of multi-view recordings, current approaches to extracting 3D representations face important limitations. Supervised pose estimation methods require extensive manual annotation of keypoints, which is labor-intensive and must be repeated for each new experimental setup or species. Mesh-based approaches such as SMAL [15] provide richer surface representations, but require a species-specific template mesh and costly per-frame optimization, limiting their scalability across species and long recordings [16, 23]. Meanwhile, recent 3D computer vision models such as VGGT [20] and E-RayZer [21] can produce dense 3D reconstructions from images in a single forward pass, but these methods are trained on generic scene datasets and perform poorly on the specialized imagery of laboratory recordings: close-up views of animals under controlled lighting from fixed camera rigs (Fig. 1). Furthermore, these models are designed for settings with dense, highly overlapping viewpoints, and devote substantial capacity to estimating unknown camera parameters—a process that can fail with limited view overlap (Fig. 6). Neither assumption holds in typical laboratory camera rigs, which use a small number of widely spaced cameras with known calibration.

We address these gaps with Beast3D, a self-supervised pretraining framework that learns 3D visual features from calibrated multi-view animal behavior videos. Given only unlabeled, synchronized multi-view images and corresponding camera parameters, Beast3D predicts a set of 3D Gaussian splats via a transformer trained to reconstruct held-out views. The model simultaneously segments the animal from the background by distilling masks from a video segmentation model, producing clean foreground representations. The self-supervised pretraining produces a flexible backbone that can be fine-tuned for multiple analytical needs. We demonstrate the quality of Beast3D’s features by comparing against state-of-the-art models across four species (mouse, rat, chickadee and human), showing substantially improved reconstructions with as few as four views (Fig. 1). We evaluate these features on three downstream tasks: (i) *novel view synthesis*, where a 3D scene constructed from a subset of views must reconstruct held-out views; (ii) *multi-view pose estimation*, where a lightweight head trained on Beast3D features predicts a sparse set of keypoints; and (iii) *neural encoding*, where Beast3D’s features predict neural activity in the mouse and chickadee datasets. In all cases Beast3D achieves competitive or superior performance compared to baselines including VGGT and DINOv3 [24]. These results establish Beast3D as a versatile framework for extracting rich 3D features from multi-view laboratory recordings, bridging the gap between general-purpose 3D computer vision and the specialized demands of behavioral neuroscience.

## 2 Related Work

### Self-supervised visual representation learning.

Self-supervised pretraining has become the dominant paradigm for learning transferable visual features. Masked image modeling approaches, exemplified by MAE [25], train a vision transformer [26] to reconstruct randomly masked pixel patches. An alternative family of methods avoids pixel-level reconstruction. I-JEPA [27] predicts latent representations of masked regions from context, avoiding the low-level pixel bias of MAE-style objectives. The DINO family [28, 29, 24] learns representations through self-distillation with momentum teachers, producing features that capture semantic structure without explicit reconstruction. These methods learn powerful 2D features but have no mechanism for reasoning about 3D structure.

### 3D scene modeling.

3D Gaussian Splatting (3DGS; [30]) represents a scene as a set of 3D Gaussians—each with its own position, shape and color parameters—rendered via differentiable rasterization, achieving high visual fidelity. A key limitation of 3DGS (as well as earlier neural radiance field approaches, [31]) is the need for per-scene optimization: each new scene requires minutes of gradient descent to fit the representation. Recent work has sought to amortize this cost through feed-forward prediction, demonstrating the inductive bias of 3D Gaussian representations can be combined with the generalization capacity of transformers to enable single-forward-pass reconstruction [32, 33, 34]. Pose Splatter [22] applies feed-forward 3DGS to multi-view animal behavior videos, using “shape carving” to initialize a voxel grid that is refined by a 3D U-Net. While Pose Splatter demonstrates accurate 3D reconstructions, it differs from Beast3D in key respects. First, Pose Splatter treats construction of a 3D latent representation as the end goal; Beast3D instead uses novel view synthesis as a pretext task to learn a general-purpose backbone whose features transfer directly to downstream tasks. Second, Pose Splatter requires foreground segmentation as a preprocessing step for shape carving, whereas Beast3D distills segmentation masks from a foundation model during training, so that inference requires only the raw multi-view images. Finally, Beast3D emphasizes multi-session training that generalizes better to new subjects.

### 3D-aware pretraining from multi-view data.

A separate line of work uses 3D geometry not as an end in itself (like 3DGS) but as an inductive bias for learning transferable visual features. RayZer [35] introduces ray-conditioned scene representations for novel view synthesis, using estimated Plücker ray coordinates [36] to encode camera geometry alongside image features. E-RayZer [21] extends this framework with an end-to-end architecture that jointly predicts camera poses and 3D Gaussians. VGGT [20] is a supervised model that predicts camera parameters, depth maps, and point clouds from a collection of images using alternating within- and across-view attention. These methods demonstrate that explicitly reasoning about 3D geometry during pretraining produces features that outperform purely 2D pretraining on geometry-sensitive downstream tasks. However, they are designed for general internet imagery where camera parameters are unknown and must be estimated jointly; models learn to do this by training on specialized datasets with dense camera coverage (often 10+ views, e.g., [37, 38]). In laboratory settings, sparse scene coverage with 3–6 cameras is more common, and accurate camera calibration is readily available. Beast3D exploits this by removing the camera parameter estimation branch entirely and instead conditions on ground-truth camera parameters. This simplification reduces the number of trainable parameters, reduces the number of required views, and allows the model to focus capacity on learning appearance and geometry features.

### Self-supervised pretraining for behavior analysis.

Selfee [39] constructs composite frames from grayscale video sequences and applies standard contrastive learning techniques, demonstrating effectiveness on downstream tasks like action segmentation and anomaly detection. Mueller et al. [40] and AnimalJEPA [41] adapt video-based joint-embedding predictive architectures to primate and mouse behavior classification, respectively. These methods produce trajectory- or clip-level representations suited to action recognition but do not target multi-camera data. Most closely related to our work, Beast [42] combines masked autoencoding with temporal contrastive learning to pretrain a vision transformer on unlabeled videos from a single experimental setup, demonstrating improvements over general-purpose baselines on downstream neural encoding and pose estimation tasks. However, Beast operates entirely in 2D image space and has no mechanism for reasoning about 3D geometry. Beast3D extends this line of work by introducing an explicit 3D inductive bias: rather than reconstructing masked image patches, it reconstructs entire held-out viewpoints through differentiable 3D Gaussian splatting. This formulation forces the representation to encode the full 3D structure of the subject—its shape, articulation, and appearance from arbitrary viewpoints—rather than merely interpolating 2D texture patterns.

## 3 Method

**Figure 2:**
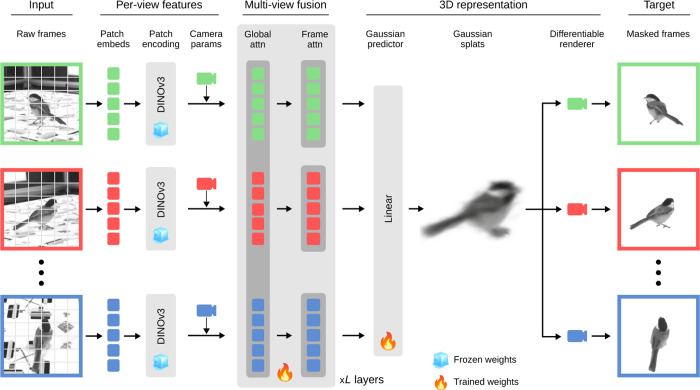
Beast3D framework. Beast3D is a masked autoencoder that uses 3D Gaussian splats as the intermediate representation. During training, one view is removed from the input and reconstructed through differentiable rendering of the 3D Gaussian splats inferred by the remaining views.

We present Beast3D, a self-supervised framework that learns 3D visual representations from multi-view animal behavior videos. Given a set of synchronized, calibrated camera views of a behaving animal, Beast3D is trained to reconstruct held-out viewpoints via differentiable 3D Gaussian splatting. The resulting encoder captures rich geometric and appearance features that transfer effectively to downstream pose estimation. Fig. 2 provides an overview of our approach.

### 3.1 Problem Setting

V synchronized cameras observe a subject from different viewpoints. At each timestep we have a set of images ${\left\{I_{v}\in {\mathbb{R}}^{H\times W\times 3}\right\}}_{v=1}^{V}$, the corresponding camera parameters, and foreground segmentation masks ${\left\{M_{v}\in {\left\{0,1\right\}}^{H\times W}\right\}}_{v=1}^{V}$ (see below). Our goal is to learn an encoder that maps images to a 3D scene representation, with no keypoint annotations. At each training step, we randomly partition the V views into a reference set R and a target set T, with |T|≥1. The model observes only the reference views and camera parameters, predicts a 3D Gaussian representation of the scene, and renders images for all views. The training signal comes from errors from the held-out target views.

### 3.2 Multi-view dataset construction

To construct training datasets we first uniformly sample one *scene* (synchronized frames across cameras) per second from each recording. To isolate the animal from the background, Beast3D learns to predict per-pixel alpha values; by supervising this channel with “ground truth” segmentation masks during training, a separate segmentation model is not required at inference time. We generate these masks offline using SAM3 [43]: an initial bounding box per view is detected automatically from a simple text prompt (“mouse”, “bird”, etc.) and propagated across subsequent frames. Running this process on every new recording at inference time would be impractical; SAM3 is computationally expensive, and mask propagation can fail on challenging frames (e.g., severe occlusions), requiring manual correction. We therefore run it once carefully during dataset construction, using a lightweight GUI to correct propagation failures, and amortize this cost into Beast3D’s trained weights.

### 3.3 Architecture

Beast3D builds on the 3DGS framework of E-RayZer [35], but replaces the learned image tokenizer and camera pose predictor with a frozen pretrained vision encoder (as in VGGT) and ground truth calibration data, respectively (Fig. 2). These choices leverage the strong visual features of modern foundation models while exploiting the accurate camera geometry available in controlled lab settings.

#### Image tokenization.

We use a frozen DINOv3 [24] ViT-B/16 as the image encoder. Each reference image $I_{v}\in {\mathbb{R}}^{H\times W\times 3}$ is first normalized with ImageNet statistics and then passed through DINOv3 to obtain patch-level features $Z_{v}\in {\mathbb{R}}^{N\times d}$, where N the number of spatial tokens and d=768 the feature dimension. We discard the [CLS] token and any register tokens, retaining only the spatial patch tokens. All DINOv3 parameters remain frozen throughout training.

#### Camera tokenization.

To provide Beast3D with geometric grounding, each pixel is associated with the ray that emanates from the camera center through that pixel’s location in 3D space. We represent each such ray in Plücker coordinates [36], a compact 6D descriptor that jointly encodes the ray’s direction in world space and its offset from the origin, and is invariant to position along the ray. The resulting Plücker map $\Pi_{v}\in {\mathbb{R}}^{H\times W\times 6}$ for view v is tokenized into patches using a linear projection to produce camera tokens $P_{v}\in {\mathbb{R}}^{N\times d}$.

#### Token fusion.

For each reference view v∈R, we augment both image and camera tokens by adding positional embeddings (fixed 2D sinusoidal positional embeddings passed through a separate ′ two-layer MLP for each modality). We concatenate these augmented tokens $Z_{v}^{\prime }$ and $P_{v}^{\prime }$ along the feature dimension and fuse them with another simple two-layer MLP.

#### Geometry transformer.

The fused tokens from all reference views are collected into a single sequence $F=\left[F_{v_{1}};\ldots ;F_{v_{\left|R\right|}}\right]\in {\mathbb{R}}^{|R|N\times d}$ and processed by a pretrained VGGT geometry transformer [20]. The transformer consists of L layers with self-attention alternating between two attention patterns: even layers apply frame attention, where each view attends only to its own tokens, while odd layers apply global attention across all views jointly. This alternating strategy allows the model to interleave 2D appearance processing with 3D multi-view aggregation.

#### 3D Gaussian prediction.

The output tokens from the geometry transformer are decoded into perpatch 3D Gaussian parameters via a linear head. Each spatial token predicts one 3D Gaussian $g_{i}$, yielding |R|⋅N Gaussians in total. Each Gaussian is described by 27 parameters that define position, shape and view-dependent color information (Appendix C).

#### Differentiable rendering.

Given the predicted 3D Gaussians ${\left\{g_{i}\right\}}_{i=1}^{|R|N}$, we render images from both reference and target views using GSplat [44]. For each view, pixel colors are computed by accumulating Gaussian contributions along each viewing ray such that Gaussians closer to the camera contribute first, with each one partially occluding those behind it. We apply an additional “frustum constraint” that culls Gaussians that fall outside the intersection of all views.

### 3.4 Training

The training loss L is computed only on the held-out target views T and combines three terms: (i) a photometric loss $L_{\ell_{2}}$ that penalizes pixel-level differences between the ground truth and rendered images; (ii) a perceptual loss $L_{\text{perc}}$ that penalizes abstract feature-level differences between ground truth and rendered images; and (iii) a mask loss $L_{\text{mask}}$ that penalizes differences between the rendered alpha channel and the SAM3 segmentation masks. The final loss is a weighted combination of each:

(1) $$L=\lambda_{\ell_{2}}L_{\ell_{2}}+\lambda_{\text{perc}}L_{\text{perc}}+\lambda_{\text{mask}}L_{\text{mask}}.$$

In our experiments we set $\lambda_{\ell_{2}}=1.0$, $\lambda_{\text{perc}}=0.2$, and $\lambda_{\text{mask}}=0.1$ across all datasets (Appendix C).

We train Beast3D using the AdamW optimizer [45] with a base learning rate of 5 × 10^−5^, weight decay of 0.05, and a cosine learning rate schedule with 15% warmup for 800 epochs. Training uses bf16 mixed precision and is distributed across 8 GPUs. During each forward pass, we randomly sample $V_{\text{ref}}=V-1$ reference views and hold out $V_{\text{tgt}}=1$ target view. The input image resolution is 256 × 256, producing N=256 patch tokens per view.

## 4 Results

We first assess 3D reconstruction capabilities through a novel view synthesis task, which tests the models’ ability to generate coherent 3D representations outside of the views it is prompted with. We then demonstrate the versatility of Beast3D through two downstream neuro-behavioral tasks: pose estimation, a dense prediction task which assesses the model’s ability to extract 3D keypoints; and neural encoding, which challenges the model to extract features that predict patterns in neural activity.

### Datasets.

We evaluate Beast3D on four datasets spanning species, environments and camera configurations (Fig. 1; Appendix A): (1) a head-fixed mouse recorded from six views (Cheese3D; [18]); (2) a freely moving rat recorded from six views (Rat7M; [2]); (3) a seed-caching chickadee recorded from six views [3]; (4) a human performing everyday tasks recorded from four views (Human3.6M [19]). Together, these datasets span both unconstrained naturalistic behavior and controlled lab settings.

### 4.1 Novel view synthesis

Novel view synthesis (NVS) is the task of rendering a scene from a camera viewpoint not present in the model’s input. This provides a direct window into the quality of the model’s internal 3D representation: a model that generates geometrically consistent, photorealistc renderings of held-out views must have learned to infer the 3D structure of the scene rather than merely memorizing 2D appearance statistics. We use NVS performance to benchmark Beast3D’s 3D reconstruction capabilities against competing methods before turning to more specialized downstream tasks.

#### Baselines.

We compare against three baselines: E-RayZer (zero-shot), the pretrained E-RayZer [21] model applied directly without any fine-tuning; E-RayZer (fine-tuned), the same model fine-tuned on each of our training datasets; and Pose Splatter [22], a feed-forward Gaussian splat model specifically designed for multi-view animal behavior recordings. We note that Pose Splatter is extensively evaluated against per-scene optimization approaches—including 3DGS [30], FSGS [46], and GO [47]—and was found to perform comparably or better overall; we therefore omit those baselines here. We also omit VGGT [20], as it produces 3D point clouds but not the accompanying color, size, and opacity information for each point required to perform the NVS task.

#### Evaluation.

E-RayZer (fine-tuned), Pose Splatter, and Beast3D are each trained on data pooled across multiple individuals. We evaluate in two complementary regimes: (1) within-subject, in which test frames are drawn from sessions included in training, but the specific test frames are held out; this matches the evaluation protocol of Pose Splatter. (2) Cross-subject, in which test frames are drawn from sessions and individuals not seen during training, assessing generalization to novel subjects. In each setting we report three standard image quality metrics. Peak signal-to-noise ratio (PSNR) quantifies pixel-level reconstruction performance relative to the dynamic range of pixel intensities. Structural similarity index measure (SSIM) captures perceptual similarity of two images by comparing luminance, contrast, and structure [48]. Learned Perceptual Image Patch Similarity (LPIPS) measures perceptual dissimilarity between image patches using deep feature activations [49]. To enable fair comparisons across baselines, all metrics are computed over foreground pixels only, identified using SAM3 segmentation masks: E-RayZer reconstructs both foreground and background by default and must be masked accordingly, while Pose Splatter uses masks internally for shape carving. Beast3D results evaluated without masks at inference time are reported in Appendix C.

#### Results.

**Figure 3:**
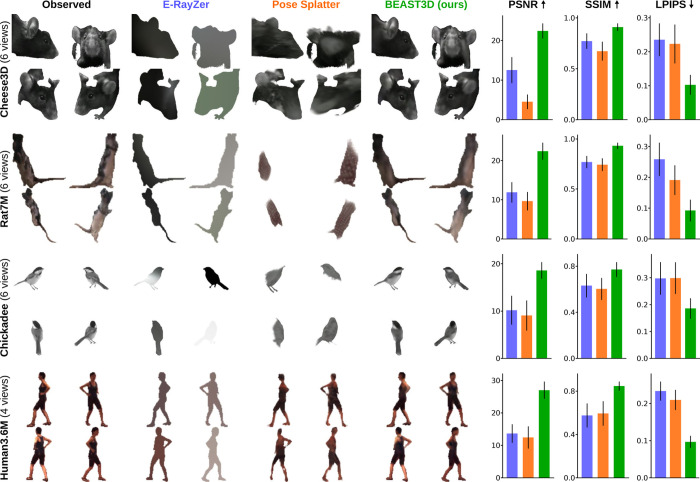
Beast3D performs high-fidelity novel view synthesis. *Left*: example within-subject, held-out target views from each dataset and the corresponding reconstructions from E-RayZer, Pose Splatter, and Beast3D, each conditioned on the remaining views from the same timestep. Reconstructions are masked by the SAM3 outputs; within these masked regions, E-RayZer often produces empty renderings, indicating that its predicted Gaussians fail to populate the subject’s location. *Right*: per-dataset PSNR, SSIM, and LPIPS (described in text); Beast3D outperforms both baselines across all metrics and datasets.

Qualitatively, Beast3D produces sharp reconstructions of held-out viewpoints that recover the subject’s silhouette and fine appearance details across all four datasets (Fig. 3; Supplementary Videos). With the ground truth masks applied, E-RayZer renderings clearly do not capture structure and appearance details of the subjects, while Pose Splatter outputs exhibit visible artifacts characteristic of shape-carving failures. Quantitatively, Beast3D improves metrics across all datasets in the within-subject evaluation setting (Fig. 3; Tables 3, 5). Across-subjects, Beast3D improves PSNR and LPIPS over the baselines on every dataset, and achieves the best SSIM on all but Chickadee, where E-RayZer is marginally higher (Tables 4, 6). E-RayZer fine-tuning yields no improvement, and occasionally degrades performance, relative to zero-shot (Appendix); we attribute this to its joint camera-pose estimation branch failing in the sparse view regime tested here (Fig. 6).

#### Ablations (Tables 3-6).

Following VGGT [20], which employs a frozen DINOv2 encoder, we use DINOv3 as our per-view image encoder. Removing DINOv3 degrades NVS performance across all datasets except for Human3.6M, demonstrating the multi-view transformer is not by itself sufficient to learn high-quality geometric representations, and benefits from strong pretrained visual features. Reinforcing this conclusion, the DINOv3 ablation *substantially* degrades pose estimation performance (Fig. 9), described in more detail in the next section. We also ablate the frustum constraint, which culls Gaussians not contained within the intersection of all viewpoints. This constraint has minimal effect on most datasets but is critical for Chickadee, where the bird is cropped from large images using a per-frame bounding box.

### 4.2 Pose Estimation

Pose estimation underpins the quantitative study of behavior in neuroscience and ethology [50, 51]. Although multi-view pose estimation is inherently 3D, most state-of-the-art pipelines process each view independently and triangulate 2D predictions into 3D as a separate post-processing step [52, 10]. In this work, we ask whether backbones that learn 3D structure during pretraining yield better pose estimators, particularly in the low-data regime typical of scientific applications. We evaluate on each dataset introduced in the novel view synthesis task.

#### Models.

All pose estimators are trained with the Lightning Pose package [53, 14]. As a strong 2D baseline we use a ViT-B/16 backbone pretrained with DINOv3 [24]; against this baseline we evaluate four backbones: Beast [42], a ViT-B/16 pretrained with masked autoencoding and temporal constrastive losses on single-view behavioral videos; VGGT [20]; E-RayZer [21]; and Beast3D.

#### Evaluation.

We adopt the limited-data evaluation protocol of the original Beast paper [42], which is designed to reflect the annotation budgets of typical labs, where exhaustive labeling is impractical. For each backbone we train three models on random subsets of only 100 labeled instances and evaluate on held-out test subjects. Because pose estimation error varies substantially across keypoints—some are more inherently ambiguous or occluded—we report results using the difficulty-stratified error curves of [53], which reveal how each method handles keypoints of varying difficulty rather than collapsing performance into a single number. “Difficulty” is quantified by the standard deviation of predictions across models and seeds for an individual keypoint on an individual frame (ensemble standard deviation, e.s.d.; higher e.s.d. indicates higher disagreement among models). Each error curve plots mean pixel error (y-axis) against an e.s.d. threshold (x-axis), where each point includes only keypoints whose e.s.d. exceeds that threshold. The leftmost point thus summarizes all keypoints, while moving rightward progressively restricts to more “difficult” ones.

#### Results.

**Figure 4:**
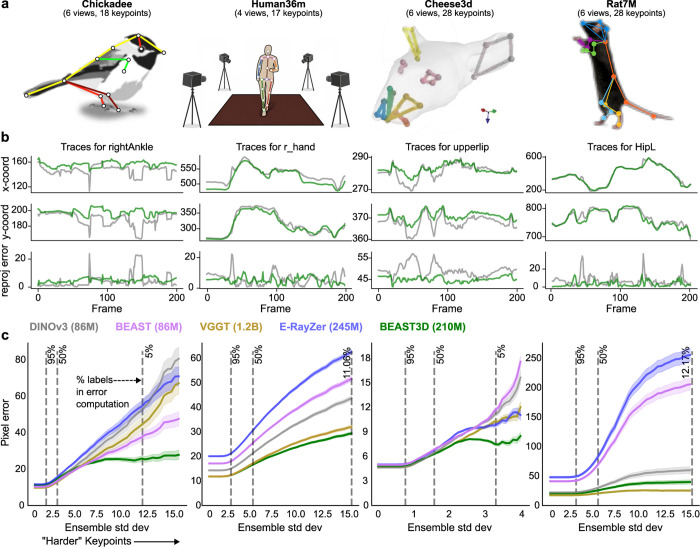
Beast3D improves pose estimation. **a:** Experimental setups and keypoint skeletons for all datasets. **b:** *Top*: representative keypoint traces from a single view. *Bottom*: corresponding 3D reprojection error for ViT-B DINOv3 (gray) and Beast3D (green). Because reprojection error leverages known camera geometry to measure agreement across views, it serves as a label-free proxy for prediction quality. Across all datasets, Beast3D traces are visibly smoother in time and incur substantially lower reprojection error. **c:** Pixel error as a function of keypoint difficulty (lower is better), computed on frames from held-out test subjects [53]. Dashed vertical lines indicate the fraction of data retained; error bands show s.e.m. across included keypoints. Results are shown for all datasets trained with subsets of 100 labeled instances.

We find that Beast3D achieves the best performance across nearly all datasets (Fig. 4), with the exception of Rat7M where VGGT—a model nearly an order of magnitude larger—performs best. Notably, Beast3D consistently outperforms Beast across all datasets, demonstrating the benefit of multi-view pretraining. However, a striking finding is that multi-view models more broadly (VGGT and E-RayZer) do not consistently outperform single-view DINOv3 and Beast baselines, despite extensive fine-tuning and hyperparameter search (Appendix G). This suggests that a multi-view architecture alone is insufficient to guarantee strong pose estimation performance; how a model is pretrained matters at least as much as its architectural capacity for 3D reasoning. Beast3D succeeds where other multi-view models fall short precisely because its novel view synthesis pretraining objective provides representations that transfer well to the pixel-level demands of pose estimation.

### 4.3 Neural encoding

A growing body of work probes the relationship between brain and behavior by predicting neural activity directly from behavior videos [54, 55, 56]. The standard approach relies on keypoints [57, 58, 59, 18], potentially missing critical behavioral features that are not well captured by tracking a sparse set of points. Beast [56] addressed this limitation by showing that learned 2D image representations outperform keypoints for neural encoding; however, these representations take the form of a single high-dimensional CLS token per frame, offering no spatial structure that would allow neuroscientists to interpret which aspects of the animal’s appearance or posture drive neural activity. Beast3D’s Gaussian splat representation is denser and more expressive than a sparse set of keypoints while remaining spatially grounded, making it more interpretable than opaque CLS tokens.

#### Datasets.

We evaluate neural encoding on two datasets: Cheese3D, with eight neurons recorded from the mouse facial motor nucleus during spontaneous behavior; and Chickadee, with 52 neurons recorded from hippocampus during seed-caching behavior. See Appendix A for details.

#### Models.

We use a variety of feature representations for neural encoding: (i) sparse 3D keypoints from pose estimation; (ii) Beast CLS tokens; (iii) Pose Splatter Gaussian splats; and (iv) Beast3D Gaussian splats. For the Gaussian splat models we we keep the centroid of each Gaussian and discard the remaining parameters that are used for rendering (e.g., opacity, color, and scale). For the keypoint-and splat-based representations we fit a PointNet transformer model [60]; for Beast we fit a temporal convolution network following [42]. See Appendix H for details.

#### Evaluation.

We split neural activity from each session into non-overlapping 2 s chunks, and randomly assign each chunk to a train, val or test split (70%/15%/15%). We tune model hyperparameters on the train/val splits, and report results on the test split using the Bits Per Spike (BPS) metric [61].

#### Results.

**Figure 5:**
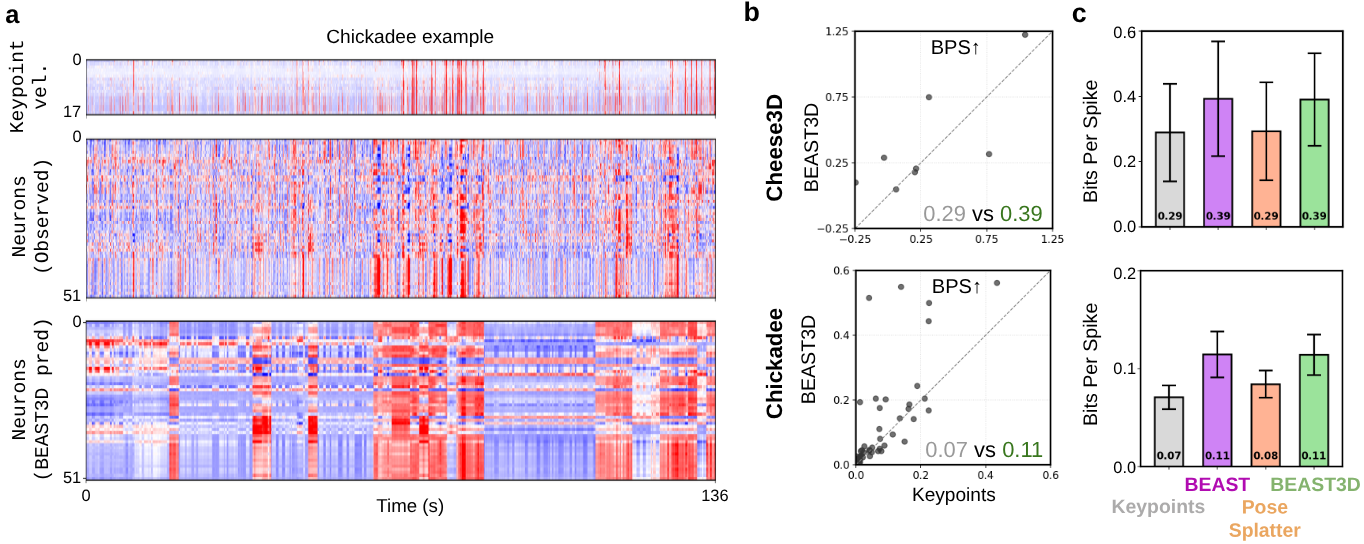
Beast3D features improve neural encoding. **a:** Example session from Chickadee. *Top*: z-scored 3D keypoint velocities. *Middle*: observed neural activity. *Bottom*: activity predicted from Beast3D Gaussian splats on held-out timepoints. **b:** Per-neuron BPS for Beast3D vs. keypoints; each dot is a neuron. Session-averaged BPS shown in bottom-right. **c:** Average BPS across keypoints, Beast, Pose Splatter, and Beast3D, with error bars showing S.E.M. across neurons.

Beast3D features outperform 3D keypoints for predicting neural activity in both datasets (Fig. 5), and per-neuron comparisons confirm that this gain is consistent across the population rather than driven by a small subset of well-predicted neurons. This demonstrates that dense 3D representations capture behavioral information that sparse keypoints discard. Beast3D also outperforms Pose Splatter, indicating that its splats capture more neurally-relevant behavioral structure. Beast3D matches the performance of Beast CLS tokens, recovering the predictive power of an opaque high-dimensional vector while preserving spatial structure. Because each Gaussian is anchored to a localized region of the subject, post-hoc analyses can ask which body parts or appearance details drive a given neuron’s activity—a question that CLS tokens cannot answer. Notably, these trends hold across both datasets despite substantial differences in species (mouse vs. bird) and brain region (facial motor nucleus vs. hippocampus).

## 5 Conclusion

We presented Beast3D, a self-supervised framework that learns dense 3D representations from multi-view animal behavior videos by reconstructing held-out viewpoints through differentiable Gaussian splatting. Across species and recording setups, we showed that Beast3D’s representations support strong 3D awareness through novel view synthesis; improve standard pose estimation pipelines in the low-annotation regime; and provide a spatially-grounded, interpretable representation for predicting neural activity. In each setting, Beast3D outperforms general-purpose 3D computer vision models such as VGGT and E-RayZer, which are designed for the dense-view, uncalibrated regime and struggle in the sparse-view laboratory setting. Beast3D also generalizes more reliably across subjects than Pose Splatter, a model tailored to multi-view animal recordings.

A current limitation of Beast3D is its compute requirements: pretraining on a single dataset takes roughly 32 hours on 8 A100 GPUs, placing it out of reach for many labs (see Appendix F for details on inference compute). Identifying architectural choices that can reduce parameter count while preserving representation quality is an important avenue for future work. The framework naturally accommodates joint training across multiple datasets, which could yield a single backbone that labs use out-of-the-box without dataset-specific pretraining. Together, these directions promise to lower the barrier to adoption and bring self-supervised 3D representation learning into broader use across the behavioral neuroscience community.

## Supplementary Material

Supplement 1
