# Supplementary material for: Beast3D: Animal behavioral analysis and neural encoding from multi-view video via Gaussian splatting
Source: ArXiv. 2026 Jun 1:arXiv:2606.02937v1. Preprint. [Version 1] (PMC13252501)
Supplement: Supplement 1 [file NIHPP2606.02937v1-supplement-1.pdf]

Supplementary Material

BEAST3D: Animal behavioral analysis and neural encoding from multi-view video via Gaussian splatting

Contents

A Datasets 16

B Dataset construction 18

C BEAST3D 20

D Baselines 22

E Novel view synthesis 24

F Inference compute cost 27

G Pose estimation 27

H Neural encoding 29

I Broader impacts 30

A Datasets

For all datasets we define a set of "In-Distribution" (InD) sessions used for training and a separate set of "Out-of-Distribution" (OOD) sessions comprising *new subjects* for evaluation. Table 1 documents the number of sessions, subjects and BEAST3D training/evaluation frames for each dataset; Appendix B details how these frames are selected. For the pose estimation task, training frames are drawn exclusively from InD sessions and test frames exclusively from OOD sessions.

A.1 Cheese3D

Behavior data. A head-fixed mouse behaves spontaneously, captured by six cameras at 100 Hz [18]. Frame sizes are 640 × 512 pixels.

For the pose estimation task, we did not have the required labeled dataset where each keypoint is labeled across all views for a given instance in time. We instead constructed a pseudo-labeled dataset from an initial set of 665 instances where each keypoint is labeled in a subset of views depending on anatomical visibility. Using this data, we trained an ensemble of three single-view transformer pose estimation models [14] using a DINOv2-pretrained ViT-B backbone (Fig. 8; step 2).

Ensemble inference. We ran inference with the ensemble across a set of full-length videos. For each time point, view and keypoint, we computed the median across the ensemble as our 2D prediction. Because training labels covered only a subset of views per keypoint, predictions in unlabeled views were systematically unreliable and received low likelihoods.

Frame filtering and triangulation. We applied a confidence filter requiring that at least two views report a median likelihood exceeding 0.6 for a given keypoint to contribute to triangulation. Keypoints

for which no view ever exceeded this threshold within a given recording session—arising from anatomical occlusion or a complete absence of training annotations across all views—were excluded from the per-frame acceptance criterion and appear as `NaN` in the final pseudo-labeled output. A frame was retained if all eligible keypoints were confidently predicted in at least two views simultaneously. For retained frames, we performed multi-view triangulation using camera parameters to recover 3D keypoint locations. These 3D estimates were then reprojected into all six views, producing geometrically consistent 2D pseudo-labels even in cameras that lacked direct confident predictions.

Frame selection. To obtain a compact yet pose-diverse pseudo-labeled set, we applied k-means clustering in the space of flattened 3D keypoints for each session. The frame closest to each cluster centroid was selected as the representative. We targeted 50-55 representative frames per session.

Reprojection error quality filtering. As a final quality control step, we computed a per-frame reprojection error as the mean Euclidean distance between the original ensemble median 2D predictions and the triangulation-derived reprojected labels, averaged over all confident keypoints and views. We discarded the worst 25% of frames by this metric. Pseudo-labels are provided for all six views per retained frame, with `NaN` entries where triangulation was not possible. The final pose estimation training/test sets consist of 450/150 instances, respectively.

Neural data. A 32-channel single-shank silicon probe was inserted into the facial motor nucleus. The probe was coated with lipophilic dyes to reveal the probe track post hoc. Recordings began at least 15 min after probe insertion to ensure recording stability. Voltage signals were acquired at 30 kHz. After the recording, single electrical pulses were delivered to all sites on the probe to induce facial movements to verify probe placement location. For this specific recording session, ear, whisker pad, nose and mouth movements were observed following electric stimulation.

Spikes were sorted into 8 well-isolated single units and binned at the downsampled video frame rate (50 Hz). The full ∼20 min session (61,697 frames) was segmented into non-overlapping 2.0 s windows of 100 frames each, yielding 616 windows. Windows were shuffled with a fixed seed and partitioned into 70% / 15% / 15% train / val / test splits (431 / 92 / 93 windows). All 8 units were retained without firing-rate thresholding.

A.2 Rat7M

A single freely moving rat behaves spontaneously in a circular arena, captured by six cameras at 120 Hz [2]. Frame sizes are 1328 × 1048 pixels.

Rat7M is a marker-based motion capture dataset with a large number of ground truth pose labels spanning multiple subjects. We retain 15 of the original keypoints in each view, omitting those tracking the head stage of the rat. Rather than retain the 30,000+ labeled instances in the original dataset, we chose to curate a smaller subset of instances matched in size with our other datasets. For each session we first filter out any instances with missing data. We then filter out potentially problematic points using skeleton distances (`ElbowR-ElbowL` distance in $[40, 60]$; `KneeR-ShinR` and `KneeL-ShinL` distances in $[10, 1000]$). We then run k-means clustering on the remaining 3D poses (using 100 clusters per session) and select one example per cluster. Finally, we performed manual inspection of the resulting labels and excluded any instances where ground truth keypoints were incorrect due to camera syncing issues. The pose estimation training/test sets consist of 455/177 instances, respectively.

The dataset is available at https://doi.org/10.6084/m9.figshare.c.5295370 under the CC-BY 4.0 license.

A.3 Chickadee

Behavior data. Freely moving chickadees engage in seed caching behavior in a large arena, captured by six cameras at 60 Hz [3]. Frame sizes vary by view but are approximately 3000 × 1500 pixels. Given the small size of the bird relative to the arena, we produced a set of cropped videos for model training. Using previously collected pose estimation labels, we trained a detector network on full resolution frames downsampled to 256 × 256 pixels to localize the bird within each frame. We computed a bounding box from the pose estimation output, and use these cropped videos for training all downstream models.

Table 1: Dataset statistics for In-Distribution (InD) and Out-of-Distribution (OOD) splits.

	In-Distribution				Out-of-Distribution		
Dataset	Subjects	Sessions	Train frames	Test frames	Subjects	Sessions	Test frames
Cheese3D [18]	6	11	44370	8382	2	4	13890
Rat7M [2]	3	5	53970	7650	2	2	21588
Chickadee [3]	6	12	64728	7650	2	4	21558
Human3.6M [19]	5	75	78596	15472	2	30	27660

For the pose estimation task, we created a cropped dataset using the ground truth labels to define a bounding box around the bird, and reshaped the cropped frames to 320 $\times$ 320 pixels. Eighteen keypoints on the chickadee's body are labeled in each view. The training/test sets consist of 433/143 instances, respectively.

Neural data. Large-scale silicon-probe recordings of one chickadee yielded 132 spike-sorted units across a multi-hour free-behavior session [3]. Spikes were binned at the video frame rate (60 Hz). To focus the encoding analysis on a behaviorally rich and neurally active window, we performed a sliding-window search over the full recording for the contiguous 15-minute interval that maximized the number of units firing at $\geq$1 Hz; this interval (54,000 frames at 60 Hz, in which 53 of 132 units exceed 1 Hz mean firing rate) is used for all subsequent encoding analyses. After cutting the videos and spike trains to this range, we segmented the spike trains into non-overlapping 2.0 s windows of 120 frames each (450 windows total), shuffled with a fixed seed, and split 70% / 15% / 15% into train / val / test (315 / 67 / 68 windows). For neural encoding, we additionally drop units whose mean spike count per training window is below 2 (i.e. $<$1 Hz), retaining 52 units; this filter is computed on training windows only and applied to all three splits to avoid data leakage.

A.4 Human3.6M

Human subjects perform a range of everyday activities captured by four synchronized cameras at 50 Hz [62, 19]. Frame sizes are 1000 $\times$ 1002 pixels.

Human3.6M is a marker-based motion capture dataset with ground truth pose labels spanning multiple subjects and 15 activity types (actions 2–16). We retain 17 keypoints in each view. Rather than retain the 3.6M labeled instances in the original dataset, similar to Rat7M we chose to curate a smaller subset of instances to benchmark the pose estimation models. Only the first subaction of each action is used; subaction variants and action 13 of subject 9 are excluded due to corrupted 3D coordinates. For each subject-action pair, we first filter out any instances with missing 3D keypoint data. We then run k-means clustering on the remaining 3D poses using 15 clusters and select the single instance closest to each cluster center, yielding up to 15 representative frames per session. We additionally require that selected frame indices are separated by at least 3 frames. The pose estimation training/test sets consist of 1125/425 instances, respectively.

The dataset is available at `http://vision.imar.ro/human3.6m` under the Human3.6M license.

B Dataset construction

All datasets are processed by a single, configurable pipeline that turns raw multi-view recordings into the calibrated *(frame, mask, camera)* tuples consumed by BEAST3D. The pipeline runs five stages in order: *cut*, *downsample*, *segment*, *assemble* and *resize*. Each stage writes its output to disk and the next stage picks it up automatically, so individual steps can be re-run without redoing earlier work. The same pipeline is invoked three times per dataset, with three different configurations, to produce the pretraining set, the in-distribution (InD) test set, and the out-of-distribution (OOD) test set.

B.1 Processing pipeline

Cut. Many of the source recordings are very long (tens of minutes to several hours) and captured at 50–120 Hz, which is far denser than what is needed for self-supervised pretraining of a static-frame model. The optional *cut* step trims each video to a user-specified inclusive frame range using `ffmpeg`'s frame-accurate `trim` filter, so that downstream steps see only the portion of the recording

that contains the behavior of interest. Whenever bounding-box CSVs accompany the videos, they are filtered and re-indexed to the same range so that bbox-frame alignment is preserved.

Downsample. The *downsample* step then resamples each video to a target frame rate via ffmpeg -vf fps=... and re-encodes with libx264 (CRF 18, audio dropped). Both *cut* and *downsample* steps process video files in parallel through a process pool to amortize cluster I/O.

Segment. For each downsampled video we run SAM3 [43] in tracking mode with a dataset-specific text prompt (e.g. mouse for Cheese3D, bird for Chickadee) to obtain a per-frame binary foreground mask. When SAM3's text grounding fails for a particular video—usually because the subject is small, occluded in the first frame, or visually atypical—the failure is recorded in failed_videos.json. We provide a small Gradio app to manually click two corners of a bounding box on the first frame of each failed video; a follow-up *retry* stage then re-runs SAM3 on those videos with the labeled boxes supplied, which bypasses text grounding and resolves all remaining failures.

Assemble. The *assemble* stage groups videos by session and camera, loads the corresponding intrinsics and extrinsics from the calibration file (Anipose toml or DeepLabCut pickle, depending on the dataset), and writes a single self-contained directory per session of the form <session>/<cam>/img{idx}.png together with a corresponding img{idx}.npy that holds the per-frame camera parameters (and bounding box, when available). The matching SAM3 mask is copied alongside as mask{idx}.png.

Resize. A final *resize* stage rescales the assembled images and masks so that the shorter side is 256 pixels, matching the input resolution expected by the DINOv3 ViT-B/16 backbone used in BEAST3D (Appendix C). Calibration intrinsics are scaled by the same factor.

B.2 Dataset splits

Pretraining set. The pretraining configuration uses the InD sessions of each dataset and aggressively downsamples each video to a low effective frame rate—1 Hz for Cheese3D and Chickadee, and a comparable rate for Rat7M and Human3.6M—under the assumption that consecutive seconds are visually near-redundant for the purpose of single-frame multi-view geometry learning. After the cut, downsample, segment, assemble, and resize stages, every retained frame becomes a training example with up to six paired views, foreground masks, and calibrated cameras. This procedure produces the training-frame counts reported in Table 1.

OOD test set. The OOD test set is constructed by re-running the exact same pipeline on a held-out set of sessions from *new subjects* that never appear during pretraining. Apart from pointing at a different input directory, the configuration is identical to the pretraining one—same target frame rate, same SAM3 prompt, same calibration loader, same resize—so the resulting frames are statistically comparable to the training frames in resolution, masking quality, and temporal density. This isolates the effect of subject identity on novel view synthesis and pose estimation performance.

InD test set. The InD test set is more delicate: it must come from the same sessions as pretraining, yet contain frames that are guaranteed to be unseen during training. We achieve this by re-running the downsample stage on the same InD videos with a *phase-shifted* frame selector. Concretely, if the source video is captured at f_{src} Hz and the pretraining target is f_{tgt} Hz, the pretraining pipeline keeps source frames at indices $\{0, S, 2S, \ldots\}$ with stride $S = \mathrm{round}(f_{\text{src}}/f_{\text{tgt}})$. The InD test pipeline shifts this lattice by an offset K with $1 \leq K < S$, keeping source frames $\{K, K + S, K + 2S, \ldots\}$. We use $K = S/2$ (e.g. $K = 50$ for Cheese3D's $f_{\text{src}} = 100$ Hz, $f_{\text{tgt}} = 1$ Hz pretraining setup), which places each test frame roughly half a sampling interval away from the nearest training frame. Because $1 \leq K < S$, the training and InD test frame sets are guaranteed to be disjoint at the source-frame level, while still being drawn from the same underlying distribution of behavior, lighting, and subjects.

C BEAST3D

For each timestep, for a dataset with V views, we have access to a set of images $\{\mathbf{I}_v \in \mathbb{R}^{H\times W\times 3}\}_{v=1}^{V}$, the corresponding camera-to-world transformations $\{\mathbf{T}_v \in \mathrm{SE}(3)\}_{v=1}^{V}$ and intrinsic parameters $\{f_x^v, f_y^v, c_x^v, c_y^v\}_{v=1}^{V}$, and foreground segmentation masks $\{\mathbf{M}_v \in \{0,1\}^{H\times W}\}_{v=1}^{V}$ via SAM3.

At each training step, we randomly partition the V views into a reference set $\mathcal{R}$ and a target set $\mathcal{T}$, with $|\mathcal{T}| \geq 1$. Our goal is to learn an encoder that maps reference views and their camera parameters to a 3D scene representation which is capable of reconstructing the target views.

C.1 Architecture details

Image tokenization. We use a frozen DINOv3 [24] ViT-B/16 as the image encoder. Each reference image $\mathbf{I}_v \in \mathbb{R}^{H\times W\times 3}$ is first normalized with ImageNet statistics and then passed through DINOv3 to obtain patch-level features:

$$\mathbf{Z}_v = \mathrm{DINOv3}(\mathbf{I}_v) \in \mathbb{R}^{N\times d}, \quad N = \left(\frac{H}{p}\right)^2, \tag{2}$$

where $p = 16$ is the patch size, N the number of spatial tokens, and $d = 768$ the feature dimension. We discard the [CLS] token and any register tokens, retaining only the spatial patch tokens. All DINOv3 parameters remain frozen throughout training.

Camera tokenization. For a pixel at location (u, w) in view v with intrinsics (f_x, f_y, c_x, c_y) and camera-to-world matrix $\mathbf{T}_v = [\mathbf{R}_v \mid \mathbf{t}_v]$, we first compute the ray direction in world coordinates:

$$\mathbf{d}_{u,w} = \mathbf{R}_v \cdot \begin{pmatrix} (u+0.5-c_x)/f_x \\ (w+0.5-c_y)/f_y \\ 1 \end{pmatrix}, \quad \hat{\mathbf{d}}_{u,w} = \frac{\mathbf{d}_{u,w}}{\|\mathbf{d}_{u,w}\|}, \tag{3}$$

and the ray origin $\mathbf{o}_v = \mathbf{t}_v$. The 6D Plücker representation is then:

$$\boldsymbol{\pi}_{u,w} = [\mathbf{o}_v \times \hat{\mathbf{d}}_{u,w};\ \hat{\mathbf{d}}_{u,w}] \in \mathbb{R}^6, \tag{4}$$

where the first three components are the moment vector and the last three are the direction.

The Plücker coordinate map $\mathbf{\Pi}_v \in \mathbb{R}^{H\times W\times 6}$ for view v is tokenized into patches using a linear projection (analogous to the image tokenizer in DINOv3) to produce camera tokens $\mathbf{P}_v \in \mathbb{R}^{N\times d}$.

Token fusion. For each reference view $v \in \mathcal{R}$, we augment both image and camera tokens with positional embeddings, which are fixed 2D sinusoidal positional embeddings passed through a separate two-layer MLP for each modality. Each MLP consists of a linear layer, SiLU activation, and another linear layer. We concatenate the augmented tokens $\mathbf{Z}_v^{'}$ and $\mathbf{P}_v^{'}$ along the feature dimension and fuse them with a two-layer MLP:

$$\mathbf{F}_v = \mathrm{MLP}_{\text{fuse}}\big([\mathbf{Z}_v^{'}\,;\,\mathbf{P}_v^{'}]\big) \in \mathbb{R}^{N\times d}, \tag{5}$$

where $\mathrm{MLP}_{\text{fuse}} : \mathbb{R}^{2d} \to \mathbb{R}^d$ consists of layer normalization, a linear layer, SiLU activation, and another linear layer.

Geometry transformer. The fused tokens from all reference views are collected into a single sequence $\mathbf{F} = [\mathbf{F}_{v_1}; \ldots; \mathbf{F}_{v_{|\mathcal{R}|}}] \in \mathbb{R}^{|\mathcal{R}|N\times d}$ and processed by a geometry transformer. Following VGGT [20], the transformer consists of L layers with QK-normalized multi-head self-attention [63] that alternate between two attention patterns:

- **Frame attention** (even layers): attention is computed independently within each view, allowing the model to reason about local 2D structure. Tokens are reshaped to $(|\mathcal{R}| \cdot B) \times N \times d$ so that each view attends only to its own tokens.
- **Global attention** (odd layers): attention spans all views jointly, enabling cross-view reasoning about 3D geometry. Tokens are reshaped to $B \times (|\mathcal{R}| \cdot N) \times d$ so that each token attends across all reference views.

This alternating strategy allows the model to interleave 2D appearance processing with 3D multi-view aggregation, balancing computational efficiency with geometric expressiveness. Layer weights are initialized with a depth-dependent standard deviation $\sigma_\ell = 0.02/\sqrt{2(\ell+1)}$ to stabilize training of deep transformers [20].

3D Gaussian prediction. The output tokens from the geometry transformer are decoded into per-patch 3D Gaussian parameters via a linear head. Each spatial token predicts one 3D Gaussian, yielding $|\mathcal{R}| \cdot N$ Gaussians in total. Specifically, the decoder predicts the following attributes per Gaussian:

$$\mathbf{g}_i = (\Delta\mathbf{x}_i,\ \mathbf{c}_i,\ \mathbf{s}_i,\ \mathbf{q}_i,\ \alpha_i), \tag{6}$$

where $\Delta\mathbf{x}_i \in \mathbb{R}^3$ is a position offset, $\mathbf{c}_i \in \mathbb{R}^{K\times 3}$ are spherical harmonic (SH) coefficients with $K = (\ell_{\max}+1)^2$ and $\ell_{\max} = 3$ for view-dependent color, $\mathbf{s}_i \in \mathbb{R}^3$ is the log-scale, $\mathbf{q}_i \in \mathbb{R}^4$ is the rotation quaternion, and $\alpha_i \in \mathbb{R}$ is the pre-sigmoid opacity. The log-scale is clamped to $[s_{\min}, s_{\max}]$ for numerical stability.

Hard pixel alignment. To anchor the predicted Gaussians in 3D space, we apply a hard pixel-alignment strategy [35]. The position offset $\Delta\mathbf{x}_i$ is first mapped to a depth value $\delta_i \in [\delta_{\text{near}}, \delta_{\text{far}}]$ via a linear range function. The final 3D position is obtained by marching along the corresponding camera ray:

$$\mathbf{x}_i = \mathbf{o}_v + \delta_i \cdot \hat{\mathbf{d}}_i, \tag{7}$$

where $\mathbf{o}_v$ is the ray origin and $\hat{\mathbf{d}}_i$ is the normalized ray direction at the spatial location of token i. This constrains each Gaussian to lie on the ray cast from its corresponding pixel, which provides a strong geometric prior and improves convergence.

C.2 Loss details

The BEAST3D loss is the weighted sum of three losses: photometric, perceptual, and mask losses.

Photometric loss. We use a foreground-weighted mean squared error between the rendered image $\hat{\mathbf{I}}_v$ and an augmented ground-truth target image $\mathbf{I}_v^*$ for each target view $v \in \mathcal{T}$:

$$\mathcal{L}_{\ell_2} = \frac{1}{|\mathcal{T}|} \sum_{v\in\mathcal{T}} \left[w_{\text{fg}} \cdot \frac{\sum(\hat{\mathbf{I}}_v - \mathbf{I}_v^*)^2 \odot \mathbf{M}_v}{\sum \mathbf{M}_v} + \frac{\sum(\hat{\mathbf{I}}_v - \mathbf{I}_v^*)^2 \odot (1-\mathbf{M}_v)}{\sum(1-\mathbf{M}_v)} \right], \tag{8}$$

where $w_{\text{fg}} = 5$ up-weights the foreground region to focus learning on the subject rather than the (augmented) background. During training, a random background color $\mathbf{b} \sim \text{Uniform}([0,1]^3)$ is composited into the initial ground truth image $\mathbf{I}_v$ via $\mathbf{I}_v^* \leftarrow \mathbf{I}_v \odot \mathbf{M}_v + \mathbf{b} \odot (1-\mathbf{M}_v)$ to prevent the model from memorizing a fixed background.

Perceptual loss. Following E-RayZer [21], we add a VGG-based perceptual loss [64] that extracts multi-scale features from a pretrained VGG-19 network:

$$\mathcal{L}_{\text{perc}} = \sum_{l=0}^{5} w_l \cdot \|\phi_l(\hat{\mathbf{I}}_v) - \phi_l(\mathbf{I}_v^*)\|_1, \tag{9}$$

where $\{\phi_l\}$ are the feature maps at selected VGG layers and $\{w_l\}$ are normalization weights. This loss encourages perceptually plausible reconstructions and reduces blurriness.

Mask loss. We supervise the rendered alpha channel $\hat{\mathbf{A}}_v$ (accumulated opacity from Gaussian splatting) against the ground-truth foreground mask:

$$\mathcal{L}_{\text{mask}} = \frac{1}{|\mathcal{T}|} \sum_{v\in\mathcal{T}} \|\hat{\mathbf{A}}_v - \mathbf{M}_v\|_2^2. \tag{10}$$

We use MSE rather than binary cross-entropy to avoid excessively large gradients in background regions, which typically occupy the majority of the image.

D Baselines

D.1 VGGT

VGGT [20] is a feed-forward 3D foundation model that, given an unordered set of multi-view images, jointly predicts per-pixel world-space points (with a per-point confidence), per-view camera extrinsics, and per-view intrinsics in a single forward pass. We use VGGT strictly as an off-the-shelf reference: we do *not* fine-tune it on any of our animal datasets and instead use the public pretrained checkpoint released by the authors[1]. At inference we resize each input view to 224×224 pixels (VGGT's training resolution), run a single forward pass over all available views with bf16 autocast, and recover (i) per-view camera extrinsics and intrinsics, and (ii) a single shared point cloud by concatenating the per-pixel world points across views. To suppress low-confidence background floaters we remove points whose confidence falls in the bottom 50% of all predicted points; the remaining points retain their per-pixel RGB colors and form the VGGT point cloud used in Fig 1. No alignment to ground-truth cameras is performed for the point cloud itself; renders shown in Fig 1 are rasterized either through the VGGT-predicted cameras directly (Cheese3D, Rat7M, Chickadee) or through the ground-truth cameras (Human3.6M) after a per-frame Procrustes alignment of the ground truth camera pose into VGGT's predicted world frame (the same alignment used for the NVS evaluation in Appendix E).

D.2 E-RayZer

E-RayZer [21] is a 3D-aware transformer that consumes multi-view images and predicts a set of per-pixel 3D Gaussians, which are then rendered into target views via Gaussian splatting[2]. Unlike VGGT, we treat E-RayZer as a fine-tunable baseline: we initialize from the public pretrained checkpoint and continue self-supervised pretraining on each of our datasets independently. Concretely, the model first tokenizes each input image with a ViT-style image tokenizer, runs a VGGT-style alternating frame/global attention encoder, and decodes per-view camera extrinsics and intrinsics from a learned camera token. These *predicted* per-view cameras are converted into Plücker ray embeddings, fused with the image tokens via an MLP, processed by a geometry transformer, and decoded into per-pixel Gaussian parameters with hard pixel alignment along the corresponding camera ray; the same predicted cameras are then used as the rendering cameras for Gaussian splatting, both during pretraining and for novel-view synthesis at inference.

The three main differences from BEAST3D are: (i) E-RayZer predicts its own cameras rather than consuming the calibrated ground truth cameras as input, and renders through those predicted cameras, whereas BEAST3D consumes and renders through ground truth cameras; (ii) E-RayZer uses a simple patch tokenizer before sending patch embeddings into the geometry transformer, whereas BEAST3D uses a frozen DINOv3 backbone as a much more powerful patch tokenizer, similar to VGGT; (iii) E-RayZer does not enforce the frustum constraint that BEAST3D uses to anchor Gaussians within the visible volume of the reference cameras; and (iv) E-RayZer is trained to reconstruct its inputs with no foreground supervision, whereas BEAST3D adds a foreground-weighted reconstruction term and a mask MSE term that supervise the rendered alpha against the SAM3 segmentation mask (Appendix C). The first three differences shift what *geometry* the model can recover, while the loss difference shifts what part of the image the model is encouraged to fit.

A practical caveat is that E-RayZer was originally pretrained with 10 views per scene split into 5 reference + 5 target, a regime that assumes dense, highly overlapping coverage of the same object (as in scene-scale datasets such as DL3DV [38]). Animal behavior recordings in our lab datasets only provide 4–6 widely separated cameras whose fields of view share a small common volume, and in this sparse-camera regime E-RayZer's pose prediction collapses — the predicted cameras are essentially uncorrelated with the true poses (Fig. 6), which propagates to the Plücker ray embeddings and prevents the geometry transformer from converging.

Fine-tuning. E-RayZer was fine-tuned for 200 epochs with AdamW (lr 5×10^{-5}, weight decay 0.05) at a global batch size of 256, retaining E-RayZer's original loss weights.

[1] https://github.com/facebookresearch/vggt

[2] https://github.com/QitaoZhao/E-RayZer

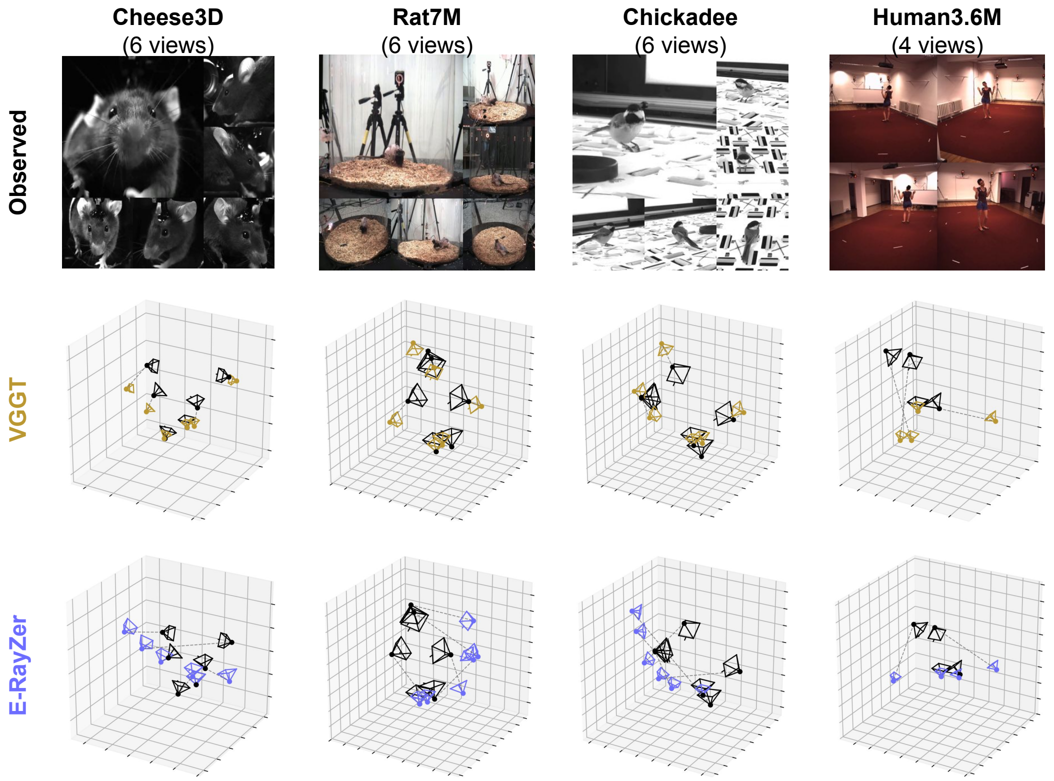

Figure 6: **Camera-pose prediction collapses in the sparse-view regime.** *Top:* representative input views. *Middle:* VGGT-predicted cameras (*colored*) paired with ground truth cameras (*black*) via dashed lines. *Bottom:* E-RayZer-predicted cameras, also paired with ground truth via dashed lines. VGGT's predictions stay close to the ground truth, but deviate more strongly on the Human3.6M dataset which only has four views. E-RayZer—which learns camera poses fully unsupervised—produces poses that are nearly uncorrelated with the true cameras under our 4–6 widely separated views.

D.3 Pose Splatter

Pose Splatter (PS) [22] is a feed-forward multi-view 3D Gaussian splatting model designed for multi-view animal datasets. Given calibrated cameras and per-view foreground masks, PS constructs a coarse voxel occupancy volume via multi-view silhouette intersection ("shape carving"), refines this representation with a 3D U-Net, and decodes occupied voxels into Gaussian parameters for differentiable rendering. Unlike generalizable reconstruction models, PS is trained per dataset.

PS differs from BEAST3D along several design dimensions that affect how 3D structure is inferred. (i) PS uses foreground masks as direct inputs to shape carving at inference time, whereas BEAST3D uses foreground masks only as supervision during training and predicts geometry from unmasked RGB images at inference time. (ii) PS initializes geometry via multi-view silhouette intersection before network refinement, introducing an explicit geometric constraint induced by mask consistency and camera alignment. BEAST3D instead predicts Gaussians directly from learned multi-view image features conditioned on camera rays. (iii) PS is trained to optimize photometric reconstruction of rendered views, while BEAST3D uses held-out-view reconstruction with an additional perceptual loss to combat vision transformer patch artifacts. These differences reflect distinct inductive biases in how geometry and appearance are represented and optimized.

Training. We use the public implementation[3] and train one model from scratch per dataset, following the provided preprocessing pipeline to produce geometry-normalized inputs with the same SAM3-generated foreground masks as those used in BEAST3D training. Models are trained for 50 epochs using Adam with a learning rate of 1×10^{-4} and batch size 1. The optimizer is used without weight decay, following the official implementation. We otherwise follow the official dataset-specific training configurations, including a voxel grid resolution of 64^3 for Cheese3D, Rat7M, and Hu-

[3] https://github.com/jackgoffinet/pose-splatter

man3.6M, and 112^3 for Chickadee, with dataset-specific cropping volumes aligned to the canonical object frame.

Evaluation. For evaluation, we compare two shape carving protocols. The *original* protocol follows the PS evaluation setup, where all available views contribute to shape carving. The *leave-one-out* (LOO) protocol matches our held-out target-view setting: for target view v, the shape carver uses only the remaining $V-1$ views at evaluation time, and the model is scored by rendering view v. Table 2 reports this comparison on the InD split with ground truth mask scoring. The original protocol improves PSNR on all datasets. We use the LOO protocol in Tables 3-6.

Table 2: **Effect of Pose Splatter carving protocol on InD GT-mask NVS.** Original uses all available views for shape carving; LOO excludes the rendered target view from the carver at evaluation time.

	Cheese3D			Human3.6M			Chickadee			Rat7M		
Protocol	PSNR↑	SSIM↑	LPIPS↓	PSNR↑	SSIM↑	LPIPS↓	PSNR↑	SSIM↑	LPIPS↓	PSNR↑	SSIM↑	LPIPS↓
Original	13.703	0.600	0.420	10.020	0.742	0.187	12.366	0.630	0.309	8.360	0.699	0.194
LOO	12.443	0.594	0.419	9.648	0.743	0.191	9.064	0.603	0.299	4.519	0.673	0.223

D.4 Beast

Beast [42] is a single-view, self-supervised pretraining procedure for animal behavior video. It pretrains a ViT-B/16 backbone with masked autoencoding (MAE) [25] on individual frames, treating each camera view as an independent image with no cross-view fusion or camera input. We pretrain one backbone per dataset using the public implementation[4], with the default recipe (mask ratio 0.75, AdamW optimizer, cosine learning rate schedule, 800 epochs); the optional InfoNCE contrastive term is disabled so that pretraining reduces to pure single-view MAE. Because Beast does not see 3D structure or cameras, it serves both as the natural 2D baseline against Beast3D and as a fine-tunable backbone for the single-view pose estimation models in Appendix G.

E Novel view synthesis

We report full novel view synthesis (NVS) results across all four datasets (Cheese3D, Rat7M, Chickadee, Human3.6M) and six methods (E-RayZer [21] zero-shot (ZS), E-RayZer fine-tuned (FT) per dataset, Pose Splatter [22], Beast3D without the frustum constraint, Beast3D without the DINOv3 backbone, and the full Beast3D). For each method–dataset pair we evaluate two regimes that match Section 4.1: the In-Distribution Test (InD Test) split, in which held-out frames come from training subjects, and the Out-of-Distribution Test (OOD Test) split, in which test frames come from unseen subjects. We also evaluate each setting under two masking conventions: foreground masked with the "ground truth" SAM3 segmentation mask (Tables 3 and 4), and foreground masked with the rendered alpha channel of each method (Tables 5 and 6). The latter additionally lets us report foreground-segmentation intersection over union (IoU) against the "ground truth" mask. Numbers are mean $\pm$ standard deviation across all evaluation frames; the best result per dataset and metric is highlighted in bold (ties at the displayed precision are bolded jointly). Cells marked "–" indicate that the corresponding evaluation is not defined for the method (E-RayZer (ZS) and E-RayZer (FT) do not produce a confident foreground alpha and so are omitted from the predicted-mask tables).

We provide example InD Test videos for each dataset, which show the synchronized camera views (*top left*), distilled foreground segmentation mask (*top right*), predicted 3D Gaussian splats for each view (*bottom left*), and a free-viewpoint orbit of the reconstructed subject demonstrating novel view synthesis capabilities (*bottom right*):

- Cheese3D
- Rat7M
- Chickadee
- Human3.6M

[4] https://github.com/paninski-lab/beast

Table 3: Novel view synthesis on the In-Distribution Test (InD Test) set (foreground masked with the GT mask). ↑ / ↓: higher / lower is better.

Dataset	Model	PSNR↑	SSIM↑	LPIPS↓
Cheese3D	E-RayZer (ZS)	13.645 ± 2.834	0.575 ± 0.110	0.467 ± 0.051
	E-RayZer (FT)	13.131 ± 2.876	0.565 ± 0.098	0.473 ± 0.057
	Pose Splatter	12.443 ± 3.404	0.594 ± 0.113	0.419 ± 0.054
	BEAST3D (no frustum)	26.578 ± 2.698	0.837 ± 0.043	0.224 ± 0.038
	BEAST3D (no DINOv3)	26.388 ± 2.457	0.832 ± 0.044	0.208 ± 0.031
	BEAST3D	$\mathbf{26.990} \pm 2.659$	$\mathbf{0.846} \pm 0.042$	$\mathbf{0.194} \pm 0.032$
Rat7M	E-RayZer (ZS)	12.536 ± 3.271	0.772 ± 0.075	0.235 ± 0.048
	E-RayZer (FT)	12.398 ± 3.314	0.764 ± 0.081	0.237 ± 0.049
	Pose Splatter	4.519 ± 1.845	0.673 ± 0.092	0.223 ± 0.057
	BEAST3D (no frustum)	22.005 ± 1.934	0.900 ± 0.038	0.113 ± 0.032
	BEAST3D (no DINOv3)	22.260 ± 1.947	0.906 ± 0.038	0.105 ± 0.030
	BEAST3D	$\mathbf{22.423} \pm 1.902$	$\mathbf{0.909} \pm 0.036$	$\mathbf{0.102} \pm 0.029$
Chickadee	E-RayZer (ZS)	10.173 ± 3.041	0.631 ± 0.102	0.298 ± 0.060
	E-RayZer (FT)	9.537 ± 3.062	0.628 ± 0.098	0.298 ± 0.058
	Pose Splatter	9.064 ± 3.172	0.603 ± 0.095	0.299 ± 0.059
	BEAST3D (no frustum)	16.760 ± 1.273	0.708 ± 0.070	0.239 ± 0.043
	BEAST3D (no DINOv3)	18.293 ± 1.783	0.767 ± 0.069	0.187 ± 0.037
	BEAST3D	$\mathbf{18.592} \pm 1.751$	$\mathbf{0.771} \pm 0.064$	$\mathbf{0.186} \pm 0.038$
Human3.6M	E-RayZer (ZS)	11.893 ± 2.591	0.769 ± 0.061	0.259 ± 0.054
	E-RayZer (FT)	13.231 ± 1.733	0.786 ± 0.053	0.258 ± 0.056
	Pose Splatter	9.648 ± 2.335	0.743 ± 0.063	0.191 ± 0.048
	BEAST3D (no frustum)	22.206 ± 2.080	0.929 ± 0.031	0.098 ± 0.036
	BEAST3D (no DINOv3)	$\mathbf{22.701} \pm 2.007$	$\mathbf{0.934} \pm 0.029$	$\mathbf{0.091} \pm 0.035$
	BEAST3D	22.386 ± 2.169	0.931 ± 0.031	0.092 ± 0.035

Table 4: Novel view synthesis on the Out-of-Distribution Test (OOD Test) set (foreground masked with the GT mask).

Dataset	Model	PSNR↑	SSIM↑	LPIPS↓
Cheese3D	E-RayZer (ZS)	12.981 ± 2.848	0.578 ± 0.101	0.467 ± 0.041
	E-RayZer (FT)	12.307 ± 2.752	0.563 ± 0.082	0.480 ± 0.047
	Pose Splatter	12.452 ± 2.873	0.592 ± 0.095	0.420 ± 0.043
	BEAST3D (no frustum)	20.175 ± 1.852	0.712 ± 0.050	0.298 ± 0.033
	BEAST3D (no DINOv3)	$\mathbf{21.220} \pm 2.060$	$\mathbf{0.732} \pm 0.049$	0.281 ± 0.025
	BEAST3D	20.736 ± 2.000	0.720 ± 0.047	$\mathbf{0.273} \pm 0.030$
Rat7M	E-RayZer (ZS)	12.322 ± 2.650	0.767 ± 0.078	0.235 ± 0.048
	E-RayZer (FT)	12.889 ± 2.682	0.751 ± 0.087	0.259 ± 0.066
	Pose Splatter	5.188 ± 2.200	0.682 ± 0.094	0.221 ± 0.058
	BEAST3D (no frustum)	$\mathbf{16.265} \pm 2.569$	$\mathbf{0.811} \pm 0.061$	0.153 ± 0.043
	BEAST3D (no DINOv3)	15.936 ± 2.510	0.810 ± 0.060	0.151 ± 0.042
	BEAST3D	16.006 ± 2.360	0.804 ± 0.061	$\mathbf{0.147} \pm 0.044$
Chickadee	E-RayZer (ZS)	10.500 ± 3.110	$\mathbf{0.667} \pm 0.105$	0.283 ± 0.063
	E-RayZer (FT)	9.454 ± 2.959	0.664 ± 0.104	0.287 ± 0.066
	Pose Splatter	8.601 ± 1.868	0.651 ± 0.090	0.253 ± 0.052
	BEAST3D (no frustum)	8.664 ± 1.618	0.523 ± 0.125	0.329 ± 0.080
	BEAST3D (no DINOv3)	9.771 ± 1.668	0.547 ± 0.109	0.297 ± 0.065
	BEAST3D	$\mathbf{13.726} \pm 1.746$	0.650 ± 0.105	$\mathbf{0.235} \pm 0.050$
Human3.6M	E-RayZer (ZS)	11.613 ± 3.623	0.724 ± 0.083	0.274 ± 0.056
	E-RayZer (FT)	12.795 ± 2.495	0.749 ± 0.064	0.275 ± 0.062
	Pose Splatter	8.560 ± 2.472	0.698 ± 0.068	0.225 ± 0.047
	BEAST3D (no frustum)	17.214 ± 1.662	0.806 ± 0.051	0.179 ± 0.037
	BEAST3D (no DINOv3)	$\mathbf{18.712} \pm 1.984$	$\mathbf{0.830} \pm 0.052$	$\mathbf{0.159} \pm 0.039$
	BEAST3D	17.107 ± 1.697	0.801 ± 0.052	0.180 ± 0.037

Table 5: Novel view synthesis and foreground segmentation on the In-Distribution Test (InD Test) set (rendered alpha as the foreground mask).

Dataset	Model	PSNR↑	SSIM↑	LPIPS↓	IoU↑
Cheese3D	Pose Splatter	15.255 ± 2.602	0.580 ± 0.109	0.440 ± 0.052	0.740 ± 0.079
	BEAST3D (no frustum)	26.087 ± 2.856	0.831 ± 0.046	0.228 ± 0.039	$\mathbf{0.933} \pm 0.059$
	BEAST3D (no DINOv3)	25.969 ± 2.649	0.827 ± 0.047	0.212 ± 0.033	0.899 ± 0.058
	BEAST3D	$\mathbf{26.623} \pm 2.838$	$\mathbf{0.842} \pm 0.044$	$\mathbf{0.197} \pm 0.033$	0.903 ± 0.058
Rat7M	Pose Splatter	9.430 ± 2.127	0.580 ± 0.103	0.377 ± 0.064	0.129 ± 0.149
	BEAST3D (no frustum)	21.956 ± 1.965	0.900 ± 0.039	0.113 ± 0.032	0.500 ± 0.087
	BEAST3D (no DINOv3)	22.160 ± 1.994	0.905 ± 0.038	0.106 ± 0.030	0.548 ± 0.097
	BEAST3D	$\mathbf{22.327} \pm 1.945$	$\mathbf{0.908} \pm 0.037$	$\mathbf{0.103} \pm 0.030$	$\mathbf{0.551} \pm 0.093$
Chickadee	Pose Splatter	10.256 ± 2.491	0.499 ± 0.134	0.395 ± 0.075	0.259 ± 0.164
	BEAST3D (no frustum)	16.640 ± 1.366	0.706 ± 0.071	0.242 ± 0.043	0.547 ± 0.055
	BEAST3D (no DINOv3)	18.029 ± 1.945	0.764 ± 0.072	0.191 ± 0.039	$\mathbf{0.692} \pm 0.085$
	BEAST3D	$\mathbf{18.314} \pm 1.910$	$\mathbf{0.768} \pm 0.066$	$\mathbf{0.189} \pm 0.039$	0.681 ± 0.081
Human3.6M	Pose Splatter	13.998 ± 1.688	0.731 ± 0.068	0.224 ± 0.064	0.581 ± 0.120
	BEAST3D (no frustum)	22.102 ± 2.126	0.928 ± 0.032	0.098 ± 0.036	0.695 ± 0.055
	BEAST3D (no DINOv3)	$\mathbf{22.624} \pm 2.046$	$\mathbf{0.933} \pm 0.029$	$\mathbf{0.091} \pm 0.035$	$\mathbf{0.718} \pm 0.056$
	BEAST3D	22.272 ± 2.223	0.930 ± 0.032	0.093 ± 0.035	0.710 ± 0.054

Table 6: Novel view synthesis and foreground segmentation on the Out-of-Distribution Test (OOD Test) set (rendered alpha as the foreground mask).

Dataset	Model	PSNR↑	SSIM↑	LPIPS↓	IoU↑
Cheese3D	Pose Splatter	14.300 ± 2.710	0.572 ± 0.095	0.447 ± 0.044	0.740 ± 0.062
	BEAST3D (no frustum)	19.569 ± 1.750	0.700 ± 0.050	0.305 ± 0.032	$\mathbf{0.886} \pm 0.039$
	BEAST3D (no DINOv3)	$\mathbf{20.607} \pm 2.068$	$\mathbf{0.720} \pm 0.051$	0.292 ± 0.026	0.874 ± 0.043
	BEAST3D	20.209 ± 1.879	0.709 ± 0.047	$\mathbf{0.280} \pm 0.031$	0.877 ± 0.044
Rat7M	Pose Splatter	9.928 ± 2.165	0.607 ± 0.100	0.356 ± 0.062	0.175 ± 0.136
	BEAST3D (no frustum)	$\mathbf{15.853} \pm 2.716$	$\mathbf{0.802} \pm 0.064$	0.170 ± 0.049	0.343 ± 0.135
	BEAST3D (no DINOv3)	15.330 ± 2.617	0.798 ± 0.062	0.174 ± 0.047	0.355 ± 0.157
	BEAST3D	15.507 ± 2.453	0.792 ± 0.065	$\mathbf{0.169} \pm 0.051$	$\mathbf{0.400} \pm 0.112$
Chickadee	Pose Splatter	9.943 ± 1.747	0.560 ± 0.105	0.355 ± 0.062	0.382 ± 0.130
	BEAST3D (no frustum)	7.933 ± 1.443	0.494 ± 0.129	0.389 ± 0.081	0.105 ± 0.080
	BEAST3D (no DINOv3)	8.779 ± 1.460	0.514 ± 0.115	0.359 ± 0.067	0.148 ± 0.074
	BEAST3D	$\mathbf{13.016} \pm 1.817$	$\mathbf{0.634} \pm 0.107$	$\mathbf{0.258} \pm 0.054$	$\mathbf{0.462} \pm 0.113$
Human3.6M	Pose Splatter	15.281 ± 1.569	0.685 ± 0.073	0.265 ± 0.064	0.562 ± 0.151
	BEAST3D (no frustum)	16.888 ± 1.731	0.794 ± 0.056	0.185 ± 0.040	0.635 ± 0.067
	BEAST3D (no DINOv3)	$\mathbf{18.427} \pm 2.018$	$\mathbf{0.822} \pm 0.055$	$\mathbf{0.163} \pm 0.041$	0.625 ± 0.068
	BEAST3D	16.741 ± 1.764	0.788 ± 0.057	0.187 ± 0.040	$\mathbf{0.640} \pm 0.068$

F Inference compute cost

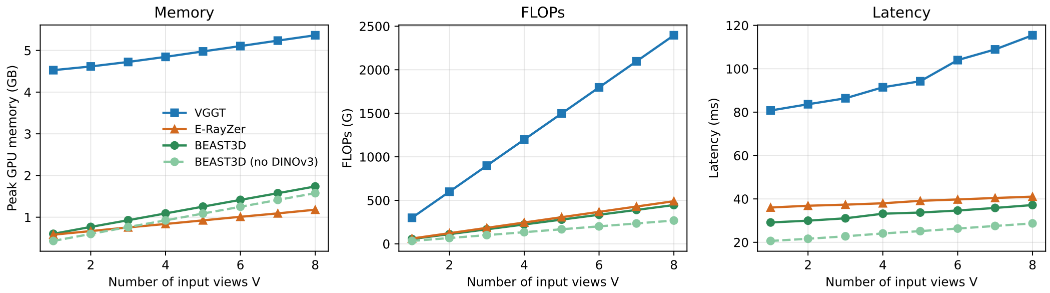

Figure 7: **Inference compute cost vs. number of input views.** Peak GPU memory (left), FLOPs (middle), and median latency over 20 timed iterations after 5 warmup (right) for VGGT, E-RayZer, and BEAST3D (with and without DINOv3), swept over $V \in \{1, \dots, 8\}$ at batch size 1, 256×256 input. Default deployment config, single GPU, bfloat16 autocast.

We benchmark inference cost of BEAST3D, VGGT, and E-RayZer as the number of input views grows (Fig. 7). All models run in their default deployment configuration on a single GPU with synthetic 256×256 inputs in bfloat16. VGGT is the most expensive along every axis (roughly $5\times$ the FLOPs and latency of the lightweight models). BEAST3D and E-RayZer have comparable compute and memory profiles, with the DINOv3 frontend in BEAST3D adding a roughly constant per-view memory and latency overhead.

G Pose estimation

G.1 Model training

We evaluate five backbone architectures spanning both single-view and 3D-aware multi-view models. All models share a common training framework built on Lightning Pose [53, 14], with architecture-specific modifications described below. For all models, backbone features are transformed into per-view keypoint heatmaps using a lightweight head consisting of transposed convolutions, following ViTPose [65], which demonstrated that ViT backbones are sufficiently expressive that simple linear/deconvolution heads are sufficient for strong pose estimation performance. Standard DLC-style augmentations [66]—including rotation, scaling, and cropping—are applied during training. Images are resized to 256×256 pixels and heatmaps are downsampled by a factor of 2, yielding 64×64 target heatmaps per keypoint per view. All models are trained for 300 epochs with the Adam optimizer and a multi-step learning rate schedule that decays the rate by a factor of 0.5 at epochs 150, 200, and 250. We employ a two-stage training strategy: backbone weights are frozen for the first 20 epochs to allow the randomly initialized prediction head to adapt to the backbone's feature space, after which the entire network is fine-tuned end-to-end. We use 95% of labeled frames for training and 5% for validation, and sample 100 labeled frames across all views per dataset for all experiments. We monitor the validation loss every 5 epochs and retain the checkpoint with the lowest validation loss as the final model.

Single-view heatmap models. The first group of methods processes each camera view independently in the same way (Fig. 8) through a shared view-agnostic backbone followed the heatmap head. We consider two backbones in this category:

- **ViT-B DINOv3**: A ViT-B pretrained with DINOv3 self-supervised learning [24], which learns semantically rich patch-level representations without explicit supervision.
- **BEAST**: A ViT-B pretrained using the BEAST self-supervised pretraining procedure on the target dataset [42], then fine-tuned for pose estimation. This two-stage approach provides dataset-specific feature initialization, and was previously shown to outperform convolution-based architectures (i.e. ResNet-50) as well as other transformer-based architectures (e.g., DINOv1 and DINOv2).

In both single-view models, per-view heatmaps are predicted independently and the final 2D keypoint locations are obtained via soft-argmax on each heatmap. These models use a training batch size of 8.

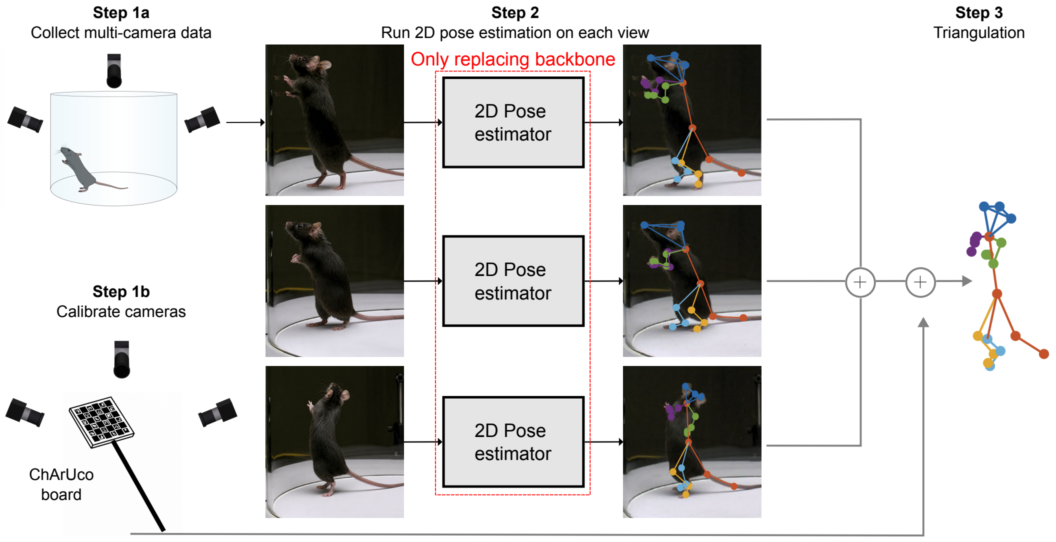

Figure 8: **Pose estimation pipeline for single-view heatmap models.** Step 1: Collect synchronized multi-camera data and calibrate cameras using a ChArUco board. Step 2: Run 2D pose estimation independently on each view, sweeping the backbone across single-view heatmap models for comparison. Step 3: Triangulate the per-view 2D predictions into 3D keypoints using the calibrated camera parameters.

Multi-view 3D-aware models. The second group of methods explicitly leverages multi-view geometry by fusing information across camera views within the model architecture. We consider three architectures:

- **VGGT**: A ViT-L backbone pretrained with DINOv2, paired with an alternating attention aggregator that performs iterative frame-level and global-level cross-view attention to fuse multi-view features. VGGT is the largest model in our comparison.
- **E-RayZer**: A 3D-aware transformer that uses a ViT-B backbone with VGGT-style cross-view attention layers to aggregate information across views. The backbone is initialized from dataset-specific E-RayZer pretrained weights.
- **BEAST3D**: Extends the BEAST pretraining paradigm to the 3D setting, using a ViT-B backbone with cross-view transformer layers. The backbone is initialized from dataset-specific BEAST3D pretrained weights.

E-RayZer and BEAST3D use a training batch size of 8 and require camera calibration parameters as additional input. For VGGT, we reduce the training batch size to 4 and employ gradient accumulation over 2 steps (effective batch size of 8) to accommodate the model's memory requirements on a single GPU.

G.2 Hyperparameter selection

For each (backbone, dataset) pair, we perform an independent learning rate sweep over five candidates spanning three orders of magnitude: $\{1\text{e}{-5}, 5\text{e}{-5}, 1\text{e}{-4}, 5\text{e}{-4}, 1\text{e}{-3}\}$. We train three models per learning rate with different random seeds controlling both the data split and heatmap head weight initialization, record the minimum validation heatmap MSE achieved during training for each seed, and average across seeds. The learning rate minimizing this averaged metric is used for all subsequent training and evaluation. Selecting per pair ensures that no method is penalized by a shared hyperparameter choice.

Table 7 reports the selected learning rate and corresponding averaged validation loss for each (backbone, dataset) pair. The selected rates vary across both backbones and datasets.

Table 7: Selected learning rates for each model–dataset pair. For each combination, we report the optimal learning rate (selected via validation heatmap MSE loss averaged over 3 seeds) and the corresponding average validation loss ($\times 10^{-3}$).

Dataset	Model	LR	Loss
Chickadee	ViT-B DINOv3	$1e^{-4}$	18.37
	E-RayZer	$1e^{-4}$	21.55
	BEAST (ViT-B MAE)	$5e^{-5}$	20.34
	BEAST3D	$1e^{-4}$	20.28
Human3.6M	ViT-B DINOv3	$5e^{-5}$	8.58
	E-RayZer	$1e^{-4}$	10.48
	BEAST (ViT-B MAE)	$1e^{-4}$	9.21
	BEAST3D	$5e^{-5}$	7.89
Cheese3D	ViT-B DINOv3	$1e^{-4}$	2.37
	E-RayZer	$1e^{-4}$	4.24
	BEAST (ViT-B MAE)	$5e^{-4}$	2.36
	BEAST3D	$5e^{-4}$	2.81
Rat-7M	ViT-B DINOv3	$1e^{-4}$	9.26
	E-RayZer	$5e^{-5}$	11.92
	BEAST (ViT-B MAE)	$1e^{-4}$	10.05
	BEAST3D	$5e^{-5}$	9.61

G.3 Additional results

We report pose estimation results using the BEAST3D ablation without the per-view DINOv3 encoder, which produces substantially worse results (Fig. 9).

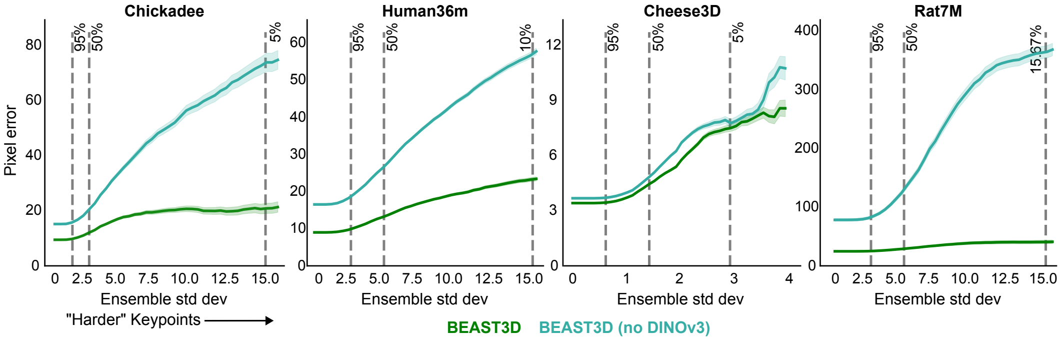

Figure 9: **Pose estimation results with DINOv3 ablation.** Figure conventions as in Fig. 4.

H Neural encoding

We assess whether the 3D representations recovered by BEAST3D carry information about simultaneously recorded neural activity by training a simple encoding model that maps a window of 3D structure to a window of per-neuron firing rates. The same model architecture and training recipe are used for both Cheese3D and Chickadee datasets; only the inputs differ. Table 8 summarizes the two electrophysiology datasets used in this section.

Per-frame 3D point clouds. For each session, we run the pretrained BEAST3D model in a held-out per-frame inference pass. For every video frame and every reference view, the geometry transformer predicts one pixel-aligned 3D Gaussian per spatial token (Eq. 7); we only keep the centroid of each Gaussian and discard the rotation, opacity, scale, and spherical-harmonic parameters that are only used for rendering. We retain only Gaussians whose predicted opacity exceeds 0.05 *and* whose corresponding pixel falls inside the SAM3 foreground mask, which removes background floaters and emphasizes the subject. For Cheese3D we additionally clip points to a fixed axis-aligned bounding box around the head and keep at most the top 8,192 points per frame ranked by opacity; for Chickadee

Table 8: Summary of the two electrophysiology datasets used for neural encoding. "Spike windows" counts the non-overlapping 2.0 s segments within the video used as encoding trials.

	**Cheese3D**	**Chickadee**
Subject / area	mouse facial motor nucleus [18]	chickadee hippocampus [3]
Views	6	6
Video FPS	50 Hz (downsampled)	60 Hz (native)
Video frames	61,697 (≈20.6 min)	54,000 (15 min)
Spike binning	50 Hz (matched to video)	60 Hz (matched to video)
Window length	2.0 s = 100 frames	2.0 s = 120 frames
Train / val / test windows	431 / 92 / 93 (total 616)	315 / 67 / 68 (total 450)
3D keypoints per frame	28	18
Point-cloud points per frame	8,192	1,024
Sorted units (raw)	8	132
Filter policy	none	1 Hz
Units used for encoding	8	52

no bounding box is applied and the full foreground point set is kept. The resulting per-frame point clouds, together with their integer frame indices, are concatenated across the session and serve as the visual input to the encoding model.

Encoding model. The encoding model is intentionally lightweight and input-agnostic so that it does not itself learn 3D structure that could compensate for a weak input. Each frame's point cloud is sub-sampled to a fixed cardinality P (P=8192 for Cheese3D, P=1024 for Chickadee), optionally centered and unit-normalized, and encoded by a per-frame PointNet [60]: a shared MLP $(3 \to 64 \to 128 \to 256)$ with batch norm and ReLU is applied to every point, followed by global max pooling and a two-layer projection to a d=256 embedding. The resulting (B, T, d) sequence of per-frame embeddings is processed by a 2-layer Transformer encoder (4 heads, GELU, pre-norm, learned positional embeddings up to length 256), and a linear readout maps each timestep to N neuron-specific rate predictions. The output is passed through a softplus to enforce positivity since we train with a Poisson negative log-likelihood loss, $\mathcal{L} = \frac{1}{BTN} \sum(\hat{\lambda}_{btn} - y_{btn} \log \hat{\lambda}_{btn})$, where y_{btn} is the observed spike count.

Training. We optimize with AdamW (weight decay 0.05, gradient clip 1.0) for 300 epochs using a cosine schedule from the configured peak learning rate down to 10^{-6} (peak 10^{-5} for Cheese3D, 10^{-3} for Chickadee, set per dataset). The training batch size is 8 for the dense Cheese3D point clouds and 32 for the smaller Chickadee point clouds. We track validation bits-per-spike (BPS) every epoch and retain the checkpoint with the highest validation BPS for test-set evaluation. Because the train / val / test windows are non-overlapping in time and disjoint at the window level, a model that only memorized the training windows cannot trivially extrapolate to test windows.

Baseline. Holding the encoding architecture and training recipe fixed, we vary only the visual input so that any difference in test BPS reflects the input representation rather than the readout. Our model uses the foreground-filtered BEAST3D point cloud, which is produced fully self-supervised: BEAST3D is pretrained without any keypoint or pose annotations, and the per-frame point cloud is read out directly from the pretrained encoder without any task-specific labels. As a baseline, we replace this input with multi-view-triangulated 3D keypoints (the EKS keypoints described in Appendix A; 28 keypoints for Cheese3D, 18 for Chickadee), which are produced by a supervised pose-estimation model trained on manually labeled keypoints in each view and then triangulated across views. This baseline therefore represents the prevailing label-dependent input modality for downstream behavioral analyses, whereas BEAST3D requires no manual labeling.

I Broader impacts

The BEAST3D framework enables more efficient extraction of meaningful information from multi-view video data, potentially accelerating behavioral neuroscience research with several beneficial outcomes. By reducing the need for extensive human labeling while improving accuracy, BEAST3D can democratize advanced video analysis capabilities for laboratories with limited resources. This efficiency could accelerate basic science discoveries that underlie advances in biomedical applications,

neurological disorder treatments, and improved understanding of brain function. While BEAST3D is developed primarily for behavioral neuroscience studies using animal subjects, the underlying technology could potentially be repurposed for human video analysis, raising several concerns:

- Surveillance capabilities: The improved ability to track behaviors could enhance surveillance technologies, potentially infringing on privacy rights if deployed without appropriate oversight.
- Bias and fairness: As with any AI system trained on specific datasets, BEAST3D-derived models may perform differently across demographic groups if applied to human subjects, potentially perpetuating biases in downstream applications.
- Resource inequality: While a pretrained BEAST3D model can improve the efficiency of downstream tasks, the computational requirements for pretraining itself may limit access to this technology for under-resourced institutions, potentially widening existing disparities in research capabilities.
